# Slow-sculpting graphene oxide/alginate gel loaded with platelet-rich plasma to promote wound healing in rats

**DOI:** 10.3389/fbioe.2024.1334087

**Published:** 2024-02-08

**Authors:** Ningjie Chen, Mengjie Li, Jincun Yang, Peng Wang, Guodong Song, Haitao Wang

**Affiliations:** ^1^ Shandong University, Jinan, Shandong, China; ^2^ Department of Burns and Plastic Surgery, Weihai Municipal Hospital, Weihai, China; ^3^ Binzhou Medical University, Binzhou, Shandong, China; ^4^ Ministry of Scientific and Technological Innovation, Yantai Hi-tech Industrial Development Zone, Yantai, Shandong, China; ^5^ Department of Burns and Orthopedic Surgery, Jinan Central Hospital, Jinan, Shandong, China

**Keywords:** slow-sculpting gel, platelet-rich plasma, wound healing, graphene oxide, alginate gel

## Abstract

Wounds, especially chronic wounds, have become an important problem that endangers human health. At present, there are many repair methods, and among them combines materials science and biology is one of the important repair methods. This study explored the preparation method, physicochemical properties, biological activity and safety of Platelet-Rich plasma (PRP)-loaded slow-sculpting graphene oxide (GO)/alginate gel, and applied it to acute full-thickness skin defect wounds in rats to observe its role in wound healing. The results show that the slow-sculpting GO/alginate gel has excellent plasticity and is suitable for a variety of irregularly shaped wounds. At the same time, its porous structure and water content can maintain the activity of platelets and their released growth factors in PRP, thereby promoting wound collagen synthesis and angiogenesis to accelerate wound healing. This indicates that the slow-sculpting GO/alginate gel is an excellent loading material for PRP, and the combination of the two may become one of the methods to promote wound repair.

## Introduction

With the intensification of the aging society and the increase of obese people, the incidence of refractory wounds such as burn/trauma, diabetic ulcers, vascular ulcers and stress injuries has increased year by year and has become a global problem that endangers human health (Collaborators et al., 2017; Dai et al., 2020; Sen, 2021). As a result, the repair and remodeling of wounds has attracted widespread attention in areas such as tissue engineering and clinical medicine (Buechler et al., 2021; Chen et al., 2021; Mascharak et al., 2021). However, wound healing is a dynamic and orderly biological process, and disturbance or interruption of any one stage will cause it to delay or not heal (Gurtner et al., 2008; Reinke and Sorg, 2012; Eming et al., 2021; Konieczny and Naik, 2021). Studies have found that a variety of functional dressings and biological therapies can promote wound healing, but the polymorphism of wounds affects the application of regular shape dressings, and the therapeutic effect of monotherapy is not satisfactory (Frykberg and Banks, 2015; Martinez-Zapata et al., 2016; Tang et al., 2022). Therefore, there is great potential for combining functional plastic biomaterials with biotherapies to rationally and effectively restore orderly wound healing processes.

Platelet-Rich plasma (PRP) as a biological therapy is rich of growth factors that can be easily produced from blood samples by centrifugation or platelet apheresis, which has been used for increasing frequency for the treatment of acute/chronic wounds (Gentile et al., 2020; Laschke and Menger, 2022). After PRP activation, the α particles in platelets release a variety of growth factors (such as PDGF, VEGF, TGF-β, EGF and FGF, *etc.*), which have the effect of promoting vascular regeneration, collagen synthesis, and involved in a specific biomolecular activity, which in turn plays an important role in acute and chronic wound healing, inflammation and pain treatment (Martinez-Zapata et al., 2016; Harrison et al., 2018; Gentile et al., 2020; Laschke and Menger, 2022). In addition, the formation of fibrin scaffolds after PRP activation can support growth factors in wound sustained release, so its biological effect is also closely related to the density of fibrin (Harrison et al., 2018; Anitua et al., 2019). However, the PRP gel scaffold is similar to the blood clot, showing a loose and porous network structure, its plasticity and adhesion are not enough to meet the needs of the wound surface, especially the chronic refractory wound surface with variable shape and different depths (Hsu et al., 2019; Irmak et al., 2020; Catanzano et al., 2021; Oneto and Etulain, 2021; Zhou et al., 2022). Therefore, through the principle of tissue engineering, loading PRP with functional materials to improve its biological activity is one of the important directions of current exploration (Qiu et al., 2016).

In recent years, sodium alginate, which has been widely used, is an anionic natural polysaccharide extracted from the cell wall and interstitium of algae, because of its gelatinity, biocompatibility and biodegradability and other advantages (Wang et al., 2018; Sanchez-Fernandez et al., 2021). In the field of biomedicine, because it has good water solubility and rich functional groups, it has excellent characteristics of structural improvement and construction of composite materials. In sodium alginate, sodium ions are exchanged with polyvalent metal cations, crosslinked to form hydrogels with good permeability, which has a significant effect on maintaining the activity of cells, enzymes, sensitive drugs, proteins and other substances (Sanchez-Fernandez et al., 2021). So, it is widely studied in the fields of tissue repair materials, biochemical drug-controlled release carriers, cell immune isolation and other tissue engineering (Kumar et al., 2019; Zhu et al., 2020; Liu et al., 2021; Rami et al., 2021; Shiny et al., 2021; Wang et al., 2021; Zhang et al., 2021). Although alginate (Alg) is currently widely used in clinical practice, and one of the most effective dressings, but there are still many deficiencies in clinical application. The shortcomings of the rapid gel process, poor mechanical strength, no biological activity, no antibacterial activity, poor stability, limited adsorption capacity and poor water resistance limit its promotion to a certain extent. Therefore, researchers try to use the composite covalent crosslinking method to modify it to solve this problem, but chemical crosslinking agents may affect its biosecurity, so physical crosslinking or ion crosslinking to improve its performance is safer and more effective, of which carbon materials are more ideal choices.

Carbon materials include graphene, graphene oxide (GO), reduced graphene oxide, carbon nanotubes, *etc.*, of which GO, as a new type of material, can combine its high specific surface area, great thermal stability, special physicochemical properties, good electronic conductivity and amazing mechanical force and other advantages, and play synergistic role with other materials (Liu et al., 2011; Xie et al., 2018). In recent years, GO-based nanocomposites have received more and more attention in many different fields, and various functional combination strategies can effectively improve the performance of GO, and give materials some properties such as sustained release, stability, photocatalytic properties, *etc.* (Wang et al., 2018; Wu et al., 2019; Joshi et al., 2020; Shen et al., 2020; Wang et al., 2020; Olmos and Gonzalez-Benito, 2021). GO surface contains a variety of oxygen-containing functional groups, with richer surface activity, good hydrophilic and biocompatible, which not only play its hydrogel good biocompatibility and moisturizing effect to promote wound healing, but also has antibacterial effect (Wu et al., 2019; Olmos and Gonzalez-Benito, 2021). Therefore, GO is widely used in antibacterial bioassays, cancer treatment, drugs and gene delivery.

In recent years, a number of studies have investigated the utilization of GO- and Alg-conjugated compounds. These compounds exhibit considerably enhanced biomechanical characteristics in comparison to Alg alone, and have been documented to possess exceptional antibacterial efficacy, as well as the ability to promote cell proliferation and facilitate drug delivery (Ciriza et al., 2015; Chen et al., 2016; Wang et al., 2019). According to the literature reviewed above, in the current study, we prepares a slow sculpting GO/Alg gel support PRP for wound repair. It is expected to provide a new idea for the preparation of wound healing materials and the promotion of PRP.

## Materials and methods

### Preparation of GO/Sodium alginate solution

The GO powder (Shenzhen Guosen Linghang Technology Co., Ltd., China) was dispersed in sterilized distilled water with a concentration of 2 mg/mL, then placed in an ultrasonic washer (Ningbo Xinzhi Biotechnology Co., Ltd., China) in room temperature for uniform dispersion for 2 h to obtain GO dispersion solution. Appropriate amount of sodium alginate powder (Shanghai Aladdin Biochemical Technology Co., Ltd., China) was added into the GO dispersion (3%wt), stirred well and placed at room temperature for later use.

### PRP preparation

For PRP preparation, male SD rats with a body weight of 260–280 g were used, after successful anesthesia with a 10% chloral hydrate (Shanghai Macklin Biochemical Co., Ltd., China), peripheral blood was drawn into a centrifuge tube containing 3.2% concentration of sodium citrate anticoagulant (peripheral blood: sodium citrate = 10:1), shaken well. Peripheral platelet concentrations were measured and PRP was prepared by the two-step centrifugation method. Centrifuge machine (Hunan Xiangyi Laboratory Instrument Development Co., Ltd., China) was used, blood was first centrifugated at 2000 rpm for 15 min at room temperature. By this step, the whole blood was divided into three layers: the upper layer contained most of the platelets and plasma while most of the red blood cells were sedimented in the bottom layer, and the middle layer (buffer layer) contained white blood cells and platelets. The upper layer and surface part of the middle layer were transferred to another sterile centrifuge tube, the transfer process should be gentle to avoid mixing with too many red blood cells. Then perform a second centrifugation at 3,000 rpm for 10 min, after the second spin, upper 2/3 was called poor platelet plasma (PPP), which contained a small number of platelets, and the lower 1/3 was PRP. 2/3 of the PPP was discarded, gently shaken the centrifuge tube to obtain final PRP. This method of PRP preparation obtained a platelet concentration of approximately 6times compared with the baseline concentration.

### Go/Alg gel loaded with PRP

The GO/Alg solution and PRP were mixed evenly with the ratio of 1:2, Added an appropriate amount of calcium carbonate powder (Shanghai Aladdin Biochemical Technology Co., Ltd., China) into the solution to insure the mass concentration of calcium carbonate was 4%, after stirring well, added glucono-delta-lactone (GDL) powder (Shanghai Aladdin Biochemical Technology Co., Ltd., China) with a the molar ratio to calcium carbonate 2.65 to the solution, and after rapid stirring, let it stand for later use.

### Gel lyophilization and electron microscopy observation

The GO/Alg gel with PRP was mixed and aliquoted into sterile EP tubes, after 1 h of standing at room temperature, the gel sample with a complete appearance and satisfactory strength was placed in the refrigerator at −80°C for 24 h, and then the frozen sample was placed into a vacuum freeze dryer (−88°C, 0.011 kPa, Labconco Co., US) for 24 h to obtain fluffy lyophilized samples. Sharply cut the middle part of the lyophilized sample for vacuum gold spraying, and then observe the microstructure of the gold-plated sample with SEM.

### Plasticity, adhesion and mechanical testing

The gel prepared by the above method was put into a 5 mL sterile bottle at room temperature, and gel formation status was observed by inverting the test tub: tilted the tube 10° every 30 s to observe the fluidity of the gel, gel was judged complete formed when no flow was observed for 10 s, and the gelation time was recorded, repeated the process for three times to take the average value (Nair et al., 2007). Different porcine tissues (skin, fat, muscle and bone) were selected to simulate their adhesion to human tissues.

The adhesive properties of the hydrogels were determined using a lap shear test at 25°C. Briefly, the specimens underwent testing using an INSTRON 5565 tensile testing apparatus (manufactured by Instron, Nor-wood, MA, United States) with a velocity of 10 mm/min until two sections of skin tissue were detached. The adhesive strength was calculated based on the maximum modulus of the adhesive area. Uniaxial tensile tests were conducted on GO/Alg, PRP, and GO/Alg/PRP scaffolds to evaluate their mechanical properties according to previous study (do Amaral et al., 2019).

### Porosity testing

The porosity was measured by the drainage method. 50 mL of absolute ethanol was added to the container, and a certain mass of sample (M1) which was already dried to the balance weight was put into the container. The container was then vacuumed circularly until no more bubbles overflow in the sample, weighed the mass of the container containing ethanol and the sample (M2). Then the sample filled with ethanol was taken out, and the remaining ethanol and volumetric bottle were weighed (M3). The porosity was calculated according to the following formula: porosity%= (M2-M3-M1)/(M2-M3) ×100%.

### Water content testing

The fully gelled hydrogel sample was immersed in PBS solution, after placing in a 37°C incubator for 24 h, removed the excess water on the surface of hydrogel with filter paper and weighed, the mass was recorded as M1. Then the hydrogel was lyophilized in a low-temperature freeze-drying machine for 24 h, and the weight of the solid after lyophilization was recorded as M2. The water content of hydrogel = (M1-M2)/M1×100%.

### Platelet fluorescence staining

PKH67 reagent was diluted 250 times through PBS solution and diluent provided in the kit (Beijing Solarbio Science and Technology CO., Ltd., China), and the prepared gel was stained *in situ* with the staining solution fully covered the gel. After standing at room temperature for 1 h, the staining solution was discarded. After fully rinsing the sample with PBS solution for 3 times, the labeling effect of platelet cells in the gel was observed under a fluorescence microscope.

### Degradation rate

The materials with the same mass of GO/Alg, PRP and GO/Alg/PRP were respectively weighed into sterile centrifuge tubes, the weighing mass was denoted as W0 and placed at room temperature (25°C, 40–50%RH). Meanwhile, the materials with the same mass of GO/Alg/PRP were placed at (37°C, 40%–50% RH). Samples were taken out and weighed at week 1,2,3,4,5,6,7 and 8, respectively. The mass of each sample was denoted as W1, and the degradation rate of the gel was calculated according to the mass change: degradation rate% = (W0-W_1_/W_0_) %. There were 3 samples in each group and the mean value was taken for statistical comparison.

### Sustained release test of PDGF

Approximately 1 mL of the freshly prepared PRP and GO/Alg/PRP solution were placed into sterile EP tubes. PRP was activated and gelatinized by hemocoagulase and calcium chloride, GO/Alg/PRP was gelatinized by adding calcium carbonate and GDL. After fully gelatinized, appropriate amount of PBS was added to completely soak the gel. Medium was changed every day; the supernatant was collected and frozen at −80° for testing. The concentration of PDGF in the supernatant was detected by rat ELISA kit (Jiangsu Meimian industrial Co., Ltd., China).

### Establishment and grouping of rat wound model

Male rats with a body weight of 260–280 g were anesthetized by intraperitoneal injection of chloral hydrate. The hair on the back of the rats was removed, and then a full-thickness skin wound with a size of 1.5 × 1.5 cm was created on the back of the rats with a scalpel. After modeling, the rats were randomly divided into 4 groups: control group, GO/Alg group (wounds were applied with GO/Alg gel without other active ingredients), PRP group (wounds were applied with homologous rat PRP) and GO/Alg/PRP group (wounds were applied with GO/Alg/PRP gel). Near-infrared irradiation was performed once a day, with 5min each time. On the 0, 3, 5, 10, and 14 days, the wound was photographed with a single-lens reflex camera, and the area of the wound was estimated using ImageJ (NIH Image, Rockville, MD, United States).

All animals were kept in a pathogen-free environment and fed *ad lib*. The procedures for care and use of animals were approved by the Ethics Committee of the Shandong University and Weihai Municipal Hospital (2021075), and all applicable institutional and governmental regulations concerning the ethical use of animals were followed.

### Histopathology and immunohistochemistry

On the 14th day of the experiment, the rats were euthanized, and the wound tissues were excised for hematoxylin-eosin (HE), Masson’s staining and CD31 immunohistochemical staining. At the same time, the heart, liver, lung and kidney were excised for further HE staining. The tissue specimens were immediately fixed with 4% paraformaldehyde (Shanghai Macklin Biochemical Co., Ltd., China) for 48 h after removal. And then dehydration, embedding and sectioning (Hua Yong, China; Leica, RM2265) were applied for follow-up inspection. The two pathologists quantitatively analyzed collagen and vascular content using ImageJ.

### Statistical analysis

SPSS22.0 software was used for data analysis, and data were expressed as mean ± standard deviation (SD). *t*-test and one-way analysis of variance (ANOVA) were used to determine statistical differences.

## Results

### Preparation and electron microscopy structure of GO/Alg/PRP

**FIGURE 1:**
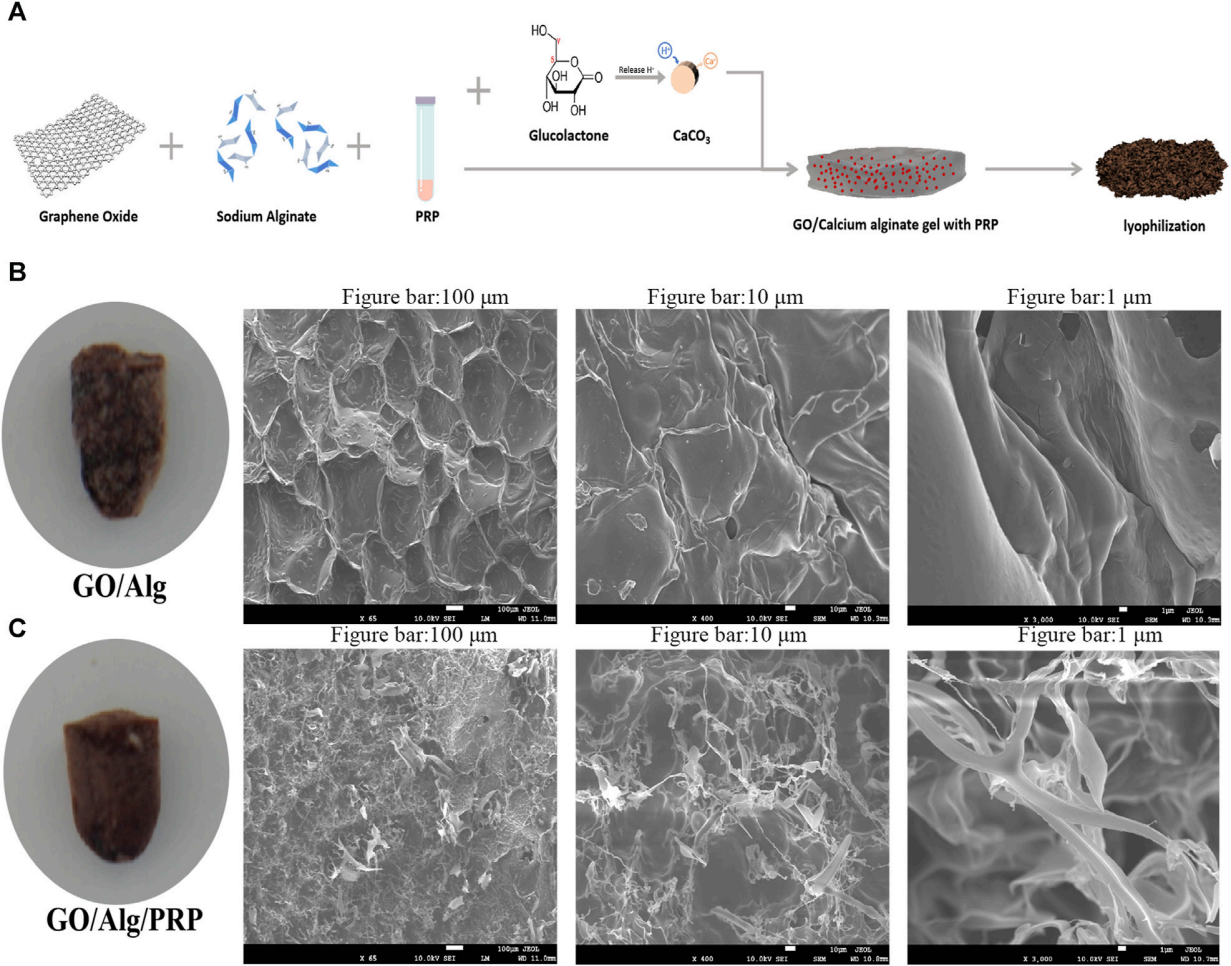
Preparation and characterization of the GO/Alg/PRP. Preparation route of the GO/Alg/PRP **(A)**. Gross appearance and SEM image of GO/Alg, GO/Alg/PRP at indicated magnification scale, and their porous structure **(B, C)**.

The preparation process of GO/Alg/PRP was shown in Figure 1A. Grossly, the general form of GO/Alg/PRP is denser than GO/Alg. Cuts the spray gold, and scans the electron microscopy to observe the microstructure. Under electron microscopy, GO/Alg and GO/Alg/PRP gels have a three-dimensional network structure, and the pore distribution is uniform and the size is relatively consistent, about 100–200 μm, indicating that GO is well dispersed, which helps GO with rich functional groups on the surface to form hydrogen bonds with hydrogels (Figure 1B); The cross-section of the pore wall is protruding, indicating that the connection with the adjacent structure is good, and there is no separation phenomenon (Figure 1B). After PRP is added, it is evenly distributed in the pores of the material, and the fibrin is cross-linked with the pore wall to form a stable three-dimensional structure (Figure 1C), and this more stable three-dimensional structure is more conducive to exerting the biological activity of PRP.

### Plasticity and adhesion of GO/Alg/PRP

**FIGURE 2:**
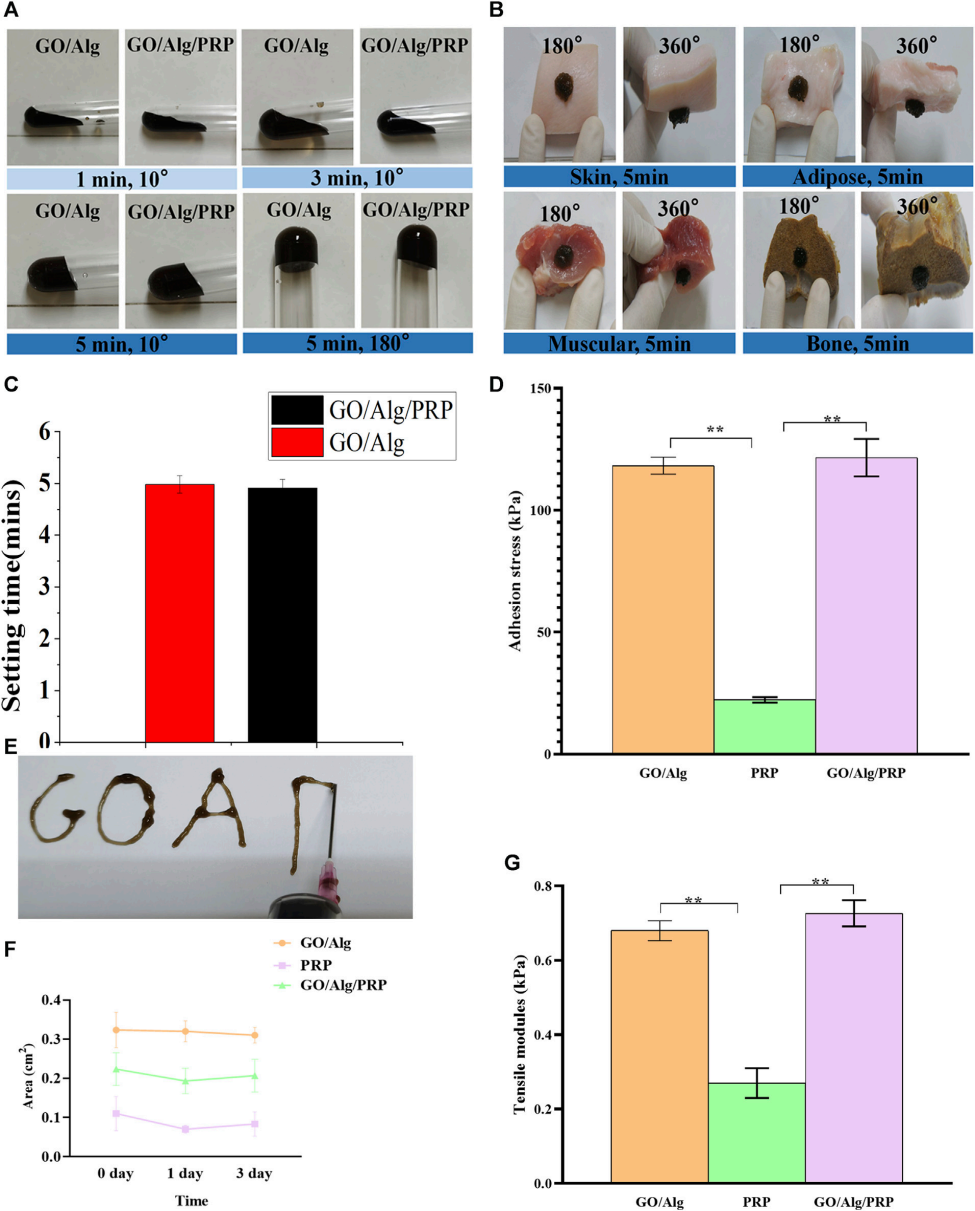
Optical photogragh of sol-gel transition of GO/Alg/PRP with 10°and 180° change on room temperature **(A)**. The GO/Alg/PRP adhered onto various biological tissues including skin, adipose, muscular and bone **(B)**. The setting time of GO/Alg and GO/Alg/PRP (*p* > 0.05), as shown in **(C)**. The adhension stress of GO/Alg/PRP **(D)**. The injectability of GO/Alg/PRP through the 18G syringe **(E)**. Area **(F)** the results showed that the GO/Alg/PRP acquired a similar shape compared to GO/Alg, as presented in tensile modulus **(G)**.

Figure 2 shows the slow plasticity and injectability of GO/Alg/PRP sols. The test tube inversion method determines the glue forming time, Go/Alg/PRP sol remains in the sol state at room temperature at 1–4 min, and the viscosity gradually increases, becoming a gel for about 5 min (Figures 2A,C), which is more suitable for clinical applications than PRP immediate gels. GO/Alg/PRP gels can be smoothly extruded through a 18G syringe at room temperature, forming specific patterns (Figure 2E), demonstrating their superior injectability.

As can be seen in Figure 2B, both GO/Alg and GO/Alg/PRP can be easily and firmly adhered to different tissues (such as skin, fat and muscle) surfaces at room temperature, while PRP’s tissue adhesion is easy to fall off from the above tissues. The adhesion stress results of the GO/Alg/PRP hydrogels indicated that the addition of GO/Alg improves the adhesion properties of the hydrogel (Figure 2D). The area of GO/Alg/PRP was larger than that of PRP group, indicated that the contractility was reduced (Figure 2F). Tensile modulus analysis revealed that PRP gel exhibits very little resistance to tension, while GO/Alg/PRP exhibits stronger resistance to tension compared to PRP and GO/Alg (Figure 2G).

### Wet environment constructed by porosity and moisture content of GO/Alg/PRP

**FIGURE 3:**
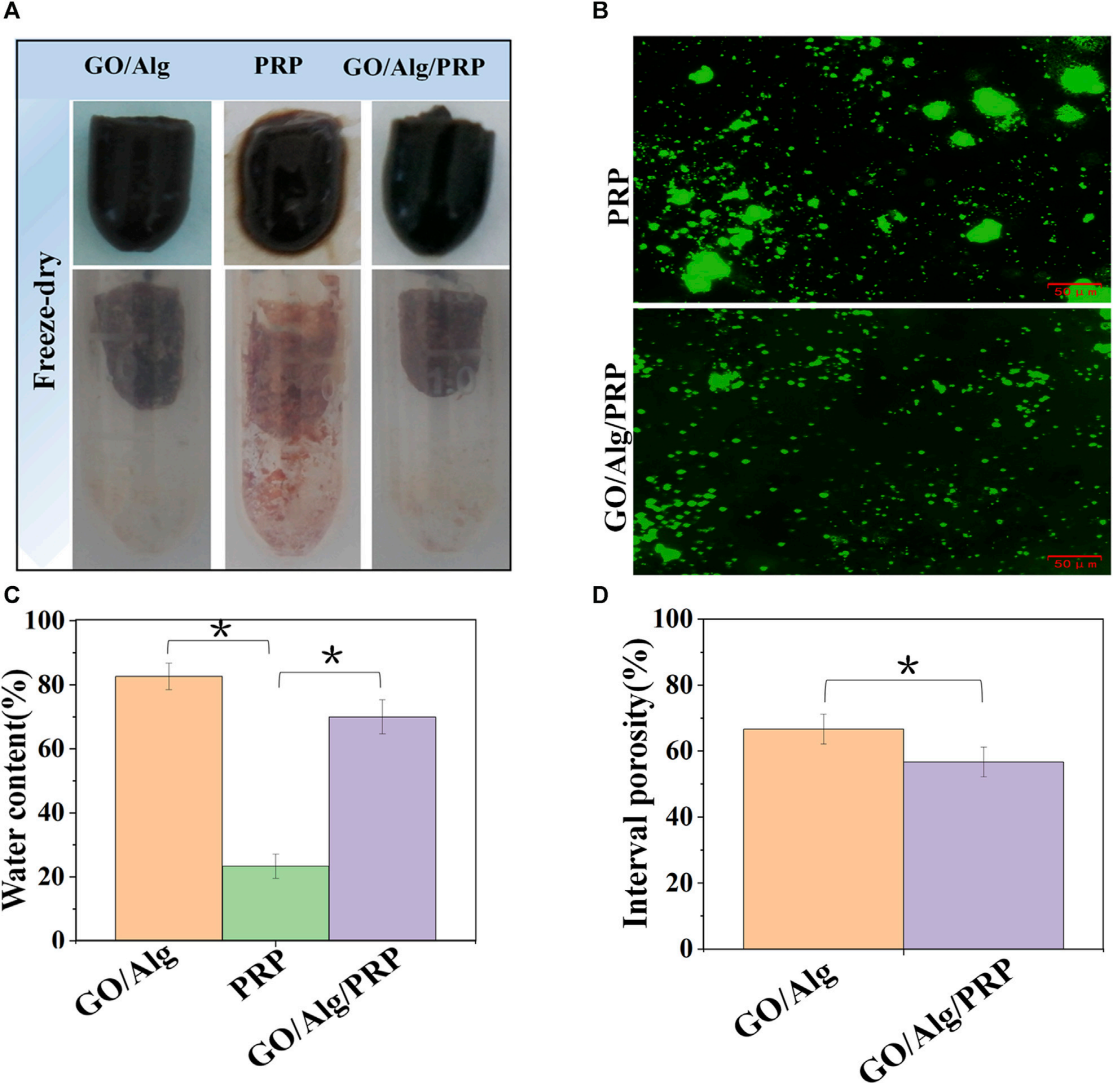
Gross appearance of GO/Alg, PRP and GO/Alg/PRP **(A)**. Immuno-fluorescence staining of platelet-rich region in PRP and GO/Alg/PRP **(B)**. Difference of water content in GO/Alg, PRP and GO/Alg/PRP, significantly more water content compared to the PRP group (^∗^ *p* < 0.05), as shown in **(C)**. Interval porosity in GO/Alg and GO/Alg/PRP, and difference were observed between GO/Alg and GO/Alg/PRP (^∗^ *p* < 0.05), as shown in **(D)**. Data were shown as mean ± SD.

As can be seen in Figure 3A, the appearance of GO/Alg and GO/Alg/PRP gels is more complete, mechanical strength is better, while PRP gels are softer and looser. After lyophilization, GO/Alg and GO/Alg/PRP gels are porous and sponge-like structures, with more pores and uniform pore size, good pore integrity, better penetration between pores, and denser than PRP gels.

The use of porous materials to act on the wound surface in order to promote its healing has always been expected by researchers, and porosity is an important factor affecting the load capacity of the material (Yao et al., 2022). Figure 3D showed that the porosity of GO/Alg decreased from (66.67 ± 4.51) % to (56.67 ± 4.51) % after adding PRP. Combined with electron microscopy images, it was found that PRP could be uniformly filled in GO/Alg gel, which further confirmed its loading effect on PRP.

From Figure 3C, it can be seen that the water content of GO/Alg and GO/Alg/PRP hydrogels is significantly higher than that of PRP gels. Immunofluorescence staining of platelets in the gel showed that the platelet aggregation rate in GO/Alg/PRP gel was lower than that in PRP gel, and platelets could flow freely in GO/Alg/PRP gel, as shown in Figure 3B.

#### Effect of GO/Alg on platelet-derived growth factor controlled release in PRP

At the same time, *in vitro* degradation experiments also found that the degradation of GO/Alg and GO/Alg/PRP gels showed a slow trend, which simulated the wet micro-environment *in vivo* and was conducive to maintaining the activity of platelets and releasing growth factors.

**FIGURE 4:**
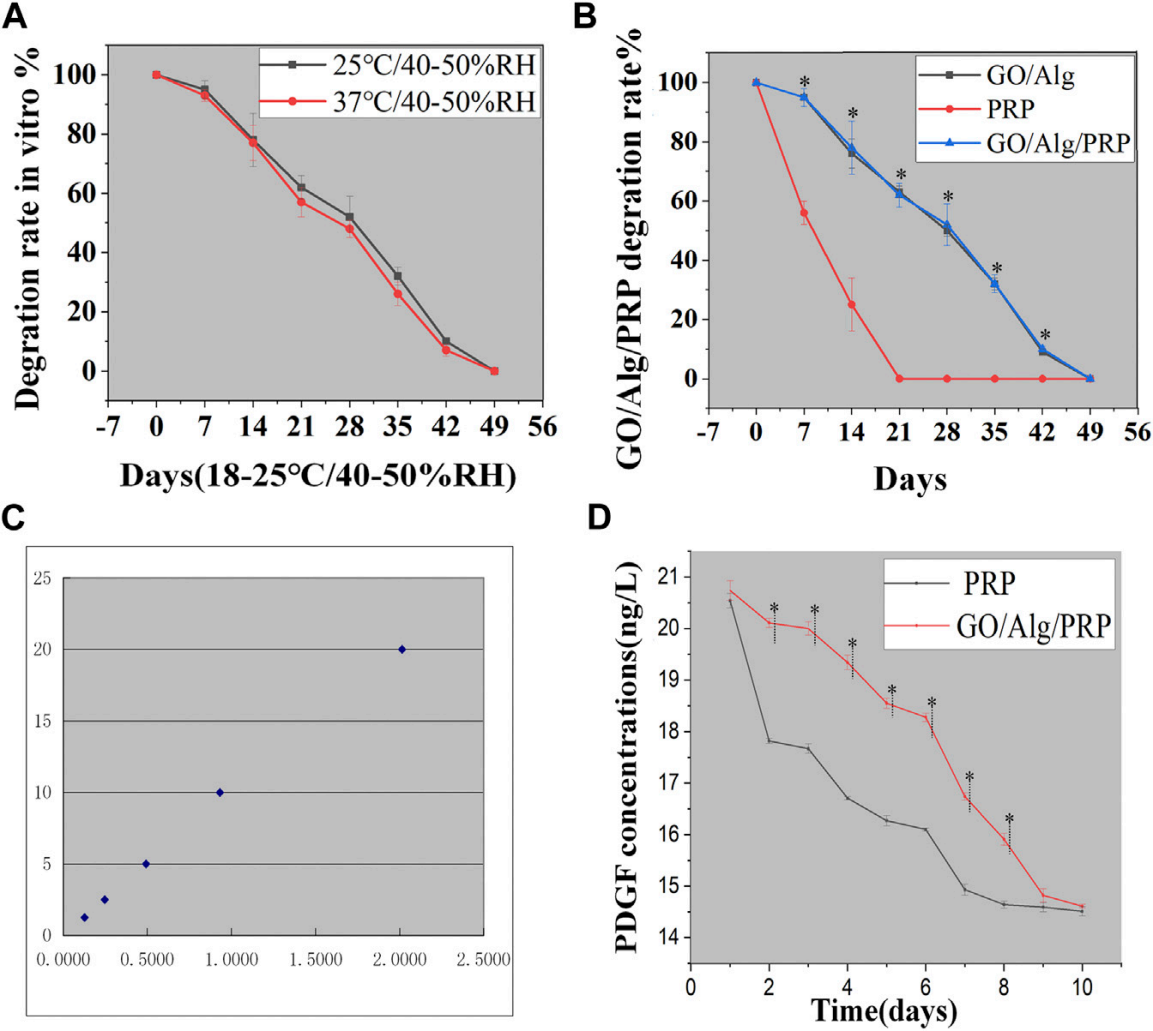
GO/Alg/PRP degradation at different temperature has no statistical difference for 7 weeks (*p* > 0.05), as shown in **(A)**. GO/Alg, PRP, and GO/Alg/PRP degradation at room temperature for 1–7 weeks (^∗^ *p* < 0.05, compared to PRP group), as shown in **(B)**. Standard curve **(C)**. PDGF slow release from PRP and GO/Alg/PRP in PBS of 37 °C, and data were shown as mean ± SD (^∗^ *p* < 0.05), as shown in **(D)**.

The degradation of GO/Alg/PRP between room temperature (2 5°C, 40%–50% RH) and body temperature (37°C, 40%–50% RH) was compared, and there was no significant difference in the degradation rate between the two observation time points, but the higher the temperature, the faster the degradation rate (Figure 4A). At the same time, GO/Alg, PRP, and GO/Alg/PRP are stored at room temperature (25°C, 40%–50% RH) under laboratory light for 1–7 weeks for degradation testing. Among them, the PRP group degraded rapidly and was basically completely degraded at 3 weeks; GO/Alg and GO/Alg/PRP degraded slowly, and there was no significant difference in vitro degradation between the two, and the degradation rate remained stable, and it was completely degraded about 7 weeks (Figure 4B).

PDGF, as a representative, plays an important role in wound healing (in collagen prolif-eration, angiogenesis and other aspects), so we choose it as a representative to compare the release kinetics of growth factors between PRP gel and GO/Alg/PRP gel. The PDGF release kinetics in PRP and GO/Alg/PRP was compared using enzyme-linked immunosorbent assay (ELISA). This study found that from day 1 to day 10, the morphology of PRP gel gradually loosened, while GO/Alg/PRP gel was intact and showed no signs of loosening except for a slight reduction in volume. According to the concentration of the standard in the ELISA kit and the absorbance measured, the standard curve was calculated. The standard concentration and the absorbance value correspond well. The standard curve equation is: Y = 0.1533 + 9.9631X. The standard curve equation has a high fitting degree with the standard curve (r = 0.991), see Figure 4C. The results showed that the release concentration of PDGF decreased gradually, and there was no statistical difference between the two on the first, ninth and 10th days. The concentration of PDGF released by GO/Alg/PRP gel was higher than that of PRP gel on the second to eighth days, and there was statistical difference between the two, as shown in Figure 4D.

### Wound General View and Healing Rate

**FIGURE 5:**
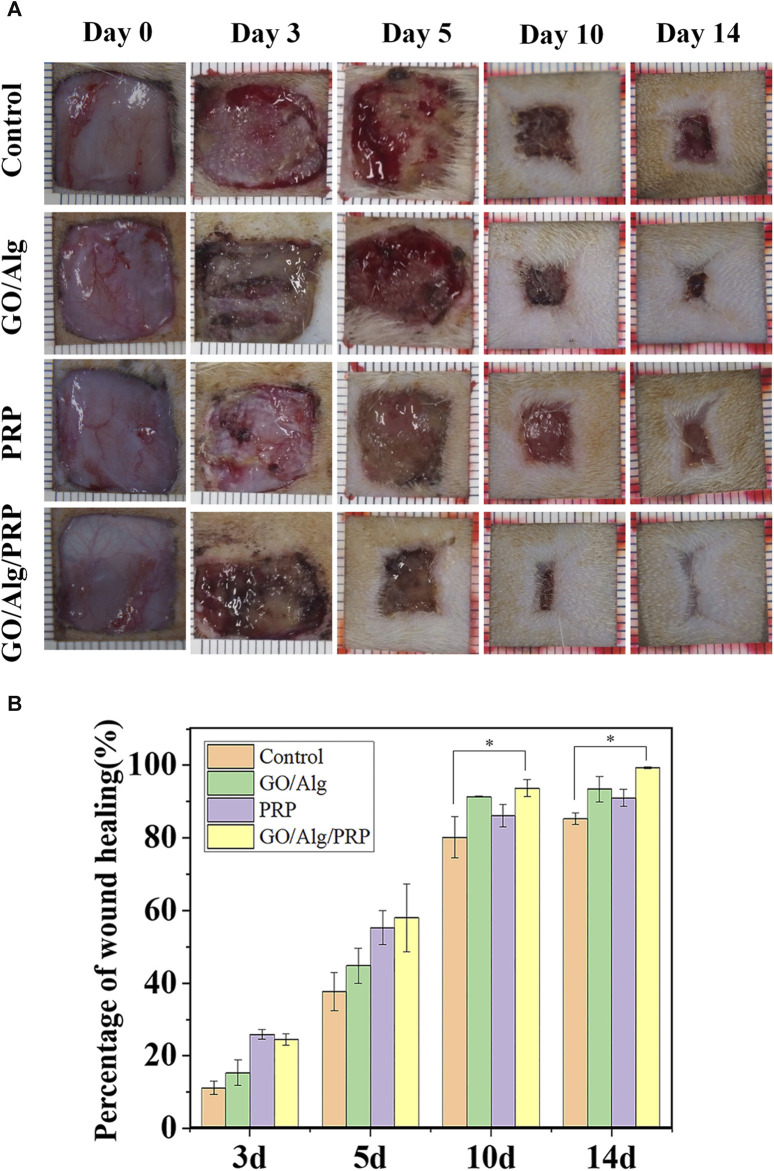
Evalution of the different groups on wound healing. Macroscopic full-thickness skin wounds images were recorded during wound healing on day 0, 3, 5, 10, and 14 **(A)**. Percentage of wound healing process **(B)**, and data were shown as mean ± SD, significantly faster wound closure was observed in GO/Alg/PRP, ^∗^ *p* < 0.05.

The external effects of the three gels were compared by *in vivo* wound experiment. Figure 5A shows the typical wound images of each group on days 0, 3, 5, 10 and 14. From the dynamic changes of wound morphology, the wounds of all groups gradually healed with time, and the GO/Alg/PRP group showed the best healing effect. Quantitative analysis of Figure 5B showed that the wound healing rate of PRP group and GO/Alg/PRP group was better than that of control group and GO/Alg group on the third and fifth day with no significant difference in wound healing rate. On the 10th and 14th days, the GO/Alg/PRP group was superior to the other three groups, and the healing speed of GO/Alg/PRP group was faster than the PRP group and the control group.

### Wound histology

**FIGURE 6:**
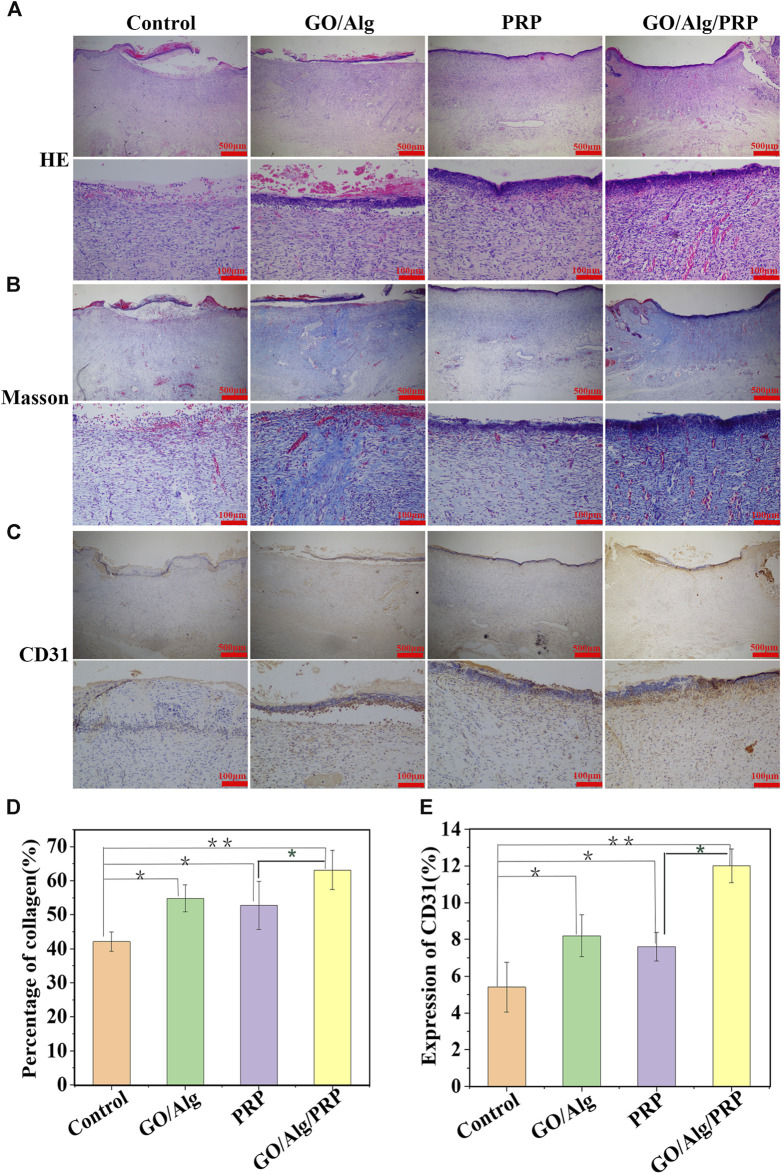
Histological appearance and analysis of the control, GO/Alg, PRP and GO/Alg/PRP group on day 14. Transmitted light images of HE-stained**(A)**, Masson’s trichrome-stained **(B)** and CD31 immunohistochemical-stained **(C)**. Significantly faster collagen synthesis **(D)** and CD31 **(E)** were observed in GO/Alg/PRP group, bars represent mean ± SD, Signifificances are presented by^∗^ *p* < 0.05,^∗∗^ *p* < 0.001.

On day 14 of the experiment, HE staining of wound tissue showed that the GO/Alg/PRP group showed better epithelial regeneration than the control group, GO/Alg group and PRP group (Figure 6A). Masson staining was used to observe collagen formation. Compared with the other three groups, the GO/Alg/PRP group not only increased collagen deposition, but also had dark blue-stained collagen directional arrangement in the tissue, and tissue remodeling was improved (Figures 6B,D). CD31 staining was used to observe angiogenesis. The brown part of the GO/Alg/PRP group was significantly higher than that of the other three groups, indicating that GO/Alg has a role in promoting vascular proliferation in wound repair (Figures 6C,E).

### Effects of metabolic rate and vital organs

**FIGURE 7:**
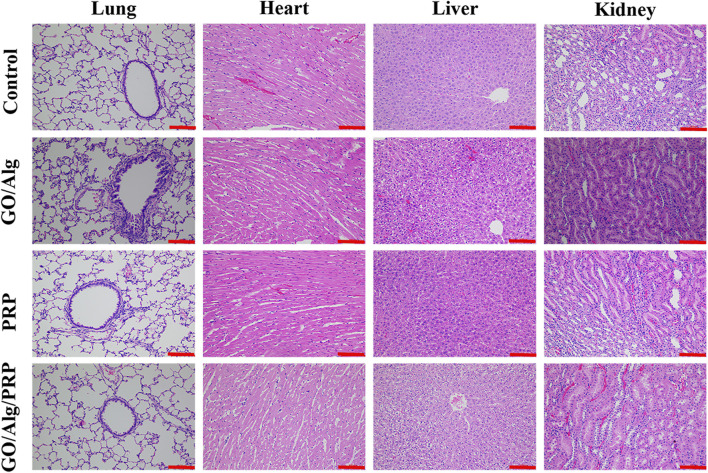
Potential organs (including lung, heart, liver and kidney) toxicity of GO/Alg/PRP with HE-stained on day 14. Scale bars are 100 μm.

Through the pathological analysis of important histopathology of the heart, lungs, liver and kidneys, the organ toxicity of GO/Alg and the presence or absence of accumulation *in vivo* were studied. On day 14 of the experiment, the stained sections of vital organs of the heart, lungs, liver and kidneys in each group showed no pathological changes, rejection inflammation, and signs of accumulation *in vivo*, indicating good biosecurity, as shown in Figure 7.

## Discussion

The results showed that the slow-sculpting GO/Alg gel has excellent plasticity and is suitable for a variety of irregularly shaped wounds. At the same time, its porous structure and water content can maintain the activity of platelets and their released growth factors in PRP, thereby promoting wound collagen synthesis and angiogenesis to accelerate wound healing.

Wound dressing materials including hydrogels, films, wafers, nanofibers, foams, topical formulations, transdermal patches, sponges and bandages were widely used in clinical. Hydrogels exhibit unique features which make them suitable wound dressings. A certain adhesion between the material and the wound can not only prevent its self-wound surf ace from falling off, but also play a role in shrinking the wound surface and promoting wound healing, which is one of the indispensable characteristics of hydrogel as a wound dressing (Li and Wang, 2011; Hu et al., 2018; Blacklow et al., 2019). By incorporating calcium ions as crosslinking agents in sodium alginate hydrogels, their mechanical properties are significantly enhanced, especially in terms of tensile strength (Wang et al., 2022). In order to enhance the plasticity and antioxidant activity of the GO/Alg gel, and then improve the biological activity and mechanical strength of PRP gel, we use gluconolactone to decompose calcium carbonate and slowly release calcium ions to produce a homogeneous calcium source, and prepares a slow sculpting gel support PRP for wound repair. Consistent with the previous studies, our results showed that the slow-sculpting GO/Alg gel has perfect porosity to act on the wound surface meanwhile the water content can create a moderate wet environment for wound healing. The excellent plasticity is suitable for a variety of irregularly shaped wounds.

The growth behavior of active substances on tissue engineering materials directly affects their tissue repair and reconstruction. Therefore, the ideal tissue engineering material must not only have a microenvironment suitable for the active substance, but also have the needs for the active substance to secrete its own extracellular matrix, nutrient infiltration, and metabolite discharge. Studies have shown that primary cells can grow albumin-secreting on three-dimensional structures for more than 6 weeks, and this function is missing during monolayer culture. Therefore, porous scaffold materials with three-dimensional microstructures play an important role in tissue engineering. Platelets in PRP are rapidly activated *in vitro* to release a variety of growth factors such as PDGF, TGF, FGF, VEGF and so on. PDGF, as a representative, plays an important role in wound healing (in collagen proliferation, angiogenesis and other aspects), so we choose it as a representative to compare the release kinetics of growth factors between PRP gel and GO/Alg/PRP gel (Hong et al., 2019; Kim et al., 2020; Laschke and Menger, 2022). Consistent with the previous studies, our study revealed that the concentration of PDGF released by GO/Alg/PRP gel was higher.

As we all know, wound healing is divided into: hemostasis, inflammation, proliferation and remodeling period, and the main risk factors affecting the delay or interruption of its healing can be roughly divided into: local factors (such as infection, hypoxia, re-injury and tissue necrosis, *etc.*), systemic diseases (such as diabetes, malnutrition and immunodeficiency, *etc.*) and pharmaceutical factors (such as the use of steroid drugs) (Martinez-Zapata et al., 2016; Zahn et al., 2017; Anitua et al., 2019; Spater et al., 2020; Laschke and Menger, 2022). In the current study, the external effects of the gels were compared by *in vivo* wound experiment. Based on the previous studies, we established a rat model of full-thickness skin defect on the back and explored the role of each group in wound healing. The wound closure rate was recorded at fixed time points from day 1–14. Wound General View and Healing Rate, HE staining and Masson results of wound tissue showed that the GO/Alg/PRP group showed better epithelial regeneration.

Both cell proliferation and migration are fundamental processes in the context of angiogenesis. Platelet endothelial cell adhesion molecule, commonly referred to as CD31, is a glycoprotein with a molecular weight of 130–140 kD. It belongs to the immunoglobulin (Ig) superfamily. The interactions between endothelial cells mediated by CD31 play a crucial role in angiogenesis. This biological process typically initiates with the proliferation of endothelial cells, which is subsequently followed by migration, adhesion, and differentiation. In the current investigation, a notable increase was observed in the GO/Alg/PRP group. This discovery implies that the sustained release of functional GO/Alg/PRP has the potential to enhance angiogenesis by expediting the proliferation and migration of endothelial cells, thereby preserving the integrity of the vascular system.

The study of GO -based materials for dressings is still in the experimental stage, and clinical applications need to clarify their metabolism and tissue and organ tox-icity. Through the pathological analysis of important histopathology of the heart, lungs, liver and kidneys, the organ toxicity of GO/Alg and the presence or absence of accumulation *in vivo* were studied. In the current study, the stained sections of vital organs of the heart, lungs, liver and kidneys in each group showed no pathological changes, rejection inflammation, and signs of accumulation *in vivo*, indicating good biosecurity of GO/Alg/PRP.

In summary, our study suggests that GO/Alg/PRP gel exhibits potential in wound repair. Nevertheless, the precise underlying mechanism remains unclear and more investigations are needed to further elucidate and clarify.

## Conclusion

The present study indicates that the slow-sculpting GO/Alg gel is an excellent loading material for PRP, and the combination of the two may become one of the methods to promote wound repair.

## Data Availability

The raw data supporting the conclusions of this article will be made available by the authors, without undue reservation.
